# Gait training assisted by multi-channel functional electrical stimulation early after stroke: study protocol for a randomized controlled trial

**DOI:** 10.1186/s13063-016-1604-x

**Published:** 2016-10-01

**Authors:** Maijke van Bloemendaal, Sicco A. Bus, Charlotte E. de Boer, Frans Nollet, Alexander C. H. Geurts, Anita Beelen

**Affiliations:** 1 Merem Rehabilitation Centre De Trappenberg, Huizen, The Netherlands; 2 Department of Rehabilitation, Academic Medical Centre, Amsterdam, The Netherlands; 3 Department of Rehabilitation, Donders Centre for Neuroscience, Radboud University Medical Centre, Nijmegen, The Netherlands

**Keywords:** Stroke, Lower Limb, Functional Electrical Stimulation, Gait, Training, Spatiotemporal Parameters

## Abstract

**Background:**

Many stroke survivors suffer from paresis of lower limb muscles, resulting in compensatory gait patterns characterised by asymmetries in spatial and temporal parameters and reduced walking capacity. Functional electrical stimulation has been used to improve walking capacity, but evidence is mostly limited to the orthotic effects of peroneal functional electrical stimulation in the chronic phase after stroke. The aim of this study is to investigate the therapeutic effects of up to 10 weeks of multi-channel functional electrical stimulation (MFES)-assisted gait training on the restoration of spatiotemporal gait symmetry and walking capacity in subacute stroke patients.

**Methods:**

In a proof-of-principle study with a randomised controlled design, 40 adult patients with walking deficits who are admitted for inpatient rehabilitation within 31 days since the onset of stroke are randomised to either MFES-assisted gait training or conventional gait training. Gait training is delivered in 30-minute sessions each workday for up to 10 weeks. The step length symmetry ratio is the primary outcome. Blinded assessors conduct outcome assessments at baseline, every 2 weeks during the intervention period, immediately post intervention and at 3-month follow-up.

**Discussion:**

This study aims to provide preliminary evidence for the feasibility and effectiveness of MFES-assisted gait rehabilitation early after stroke. Results will inform the design of a larger multi-centre trial.

**Trial registration:**

This trial is registered at the Netherlands Trial Register (number NTR4762, registered 28 August 2014)

**Electronic supplementary material:**

The online version of this article (doi:10.1186/s13063-016-1604-x) contains supplementary material, which is available to authorized users.

## Background

Regaining independent gait is considered one of the primary goals in stroke rehabilitation [1–3]. In the early phase after stroke, the musculature of the affected side is often paretic or even paralytic. As a consequence, compensatory gait patterns characterised by asymmetries in spatial and temporal parameters may arise that tend to be persistent, even in patients who show substantial restoration of paretic leg motor control, perhaps due to mechanisms related to ‘learned non-use’ as has been described for the upper extremity [4]. These compensatory gait patterns are less energy-efficient and may negatively affect balance control leading to an increased risk of falls and injury as well as to limitations in functional mobility [5–8]. Furthermore, they may cause secondary complications, such as muscle shortening and joint deformation [6]. Restoration of gait symmetry can be accomplished by motor relearning and neuroplasticity, for which highly intensive, repetitive and task-specific training is essential in the early rehabilitation phase after stroke [9, 10]. The use of functional electrical stimulation (FES) timed to the gait cycle in the early phase after stroke may improve gait symmetry by enhancing neuroplasticity, preventing secondary complications, and by supporting the acquisition of an adequate compensatory strategy. Although the orthotic effects of peroneal FES (PFES) have been established, the therapeutic effect of PFES in the subacute phase has been scarcely investigated [11–19]. Furthermore, PFES assists the ankle dorsiflexion movement only during the swing phase and early stance phase of gait and does not support the more proximal movements of the lower limb. Several studies have shown that strength and range of motion of the knee flexors and extensors are associated with gait performance [20–22]. Thus, multi-channel FES (MFES) of the distal and proximal parts of the lower limb may be more effective in normalising the gait pattern by compensating for thigh and dorsiflexor muscle weakness. There is preliminary evidence of a positive therapeutic effect of MFES in early stroke rehabilitation on balance control and mobility [23–25]. However, it remains unclear whether MFES is effective for the restoration of gait symmetry. Furthermore, it remains unclear whether it is feasible to implement MFES in functional gait training including pre-gait activities. Due to the limited evidence of MFES-assisted gait training during early stroke rehabilitation we designed a proof-of-principle study. The aim of this study is to examine the feasibility and preliminary efficacy of MFES-assisted gait training on gait symmetry and walking capacity in patients in the subacute phase after stroke during their inpatient rehabilitation. We hypothesise that MFES-assisted gait training for maximally 10 weeks in the early phase after stroke is feasible and improves the step length symmetry compared to conventional gait training. I﻿﻿n this paper we describe the protocol of our study according to the SPIRIT guidelin﻿es (Additional file 1).

## Methods

### Design

**Fig. 1:**
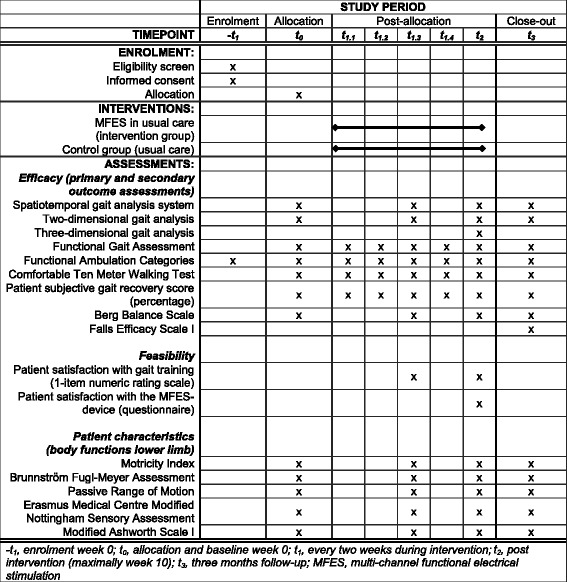
Schedule of enrolment, interventions and assessments

A prospective, assessor-blinded, single-centre, proof-of-principle study with a randomised controlled two-armed parallel design is being conducted. Forty participants with gait impairments in the subacute phase after stroke who are referred for inpatient rehabilitation are randomly assigned in a 1:1 ratio to either an intervention group, receiving MFES-assisted gait training, or a control group, receiving gait training as usual. The intervention period lasts 10 weeks or until discharge from inpatient rehabilitation, whichever is sooner. Outcomes will be assessed every 2 weeks during the 10-week intervention period as well as after a 3-month follow-up period (Fig. 1).

### Ethics

The study protocol has been approved by the Medical Ethics Committee (MEC) of the Academic Medical Centre Amsterdam (protocol number NL50002.018.14). Any changes to the study protocol or study procedures will be reviewed and approved by the MEC and communicated to relevant parties. A Dutch rehabilitation centre (Merem Rehabilitation Centre De Trappenberg in Huizen) granted approval to include and train participants. The study has been registered at the Netherlands Trial Register (number NTR4762, registered 28 August 2014). Additional file 2 provides an overview of the trial registration data.

### Participants

Participants are recruited at the rehabilitation centre. All stroke survivors admitted for inpatient rehabilitation are screened for eligibility by their physiatrist. Inclusion criteria are: (1) a clinical diagnosis of stroke (diagnostic criteria according to the World Health Organization definition) [26]; (2) within 31 days since stroke onset; (3) age between 18 and 80 years; (4) indication for gait training (according to the treating physiatrist); (5) sufficient capacity to stand between parallel bars with or without physical assistance and able to walk with aids and physical assistance from one physical therapist (Functional Ambulation Categories [FAC] score ≥1); and (6) passive range of motion (PROM) upon ankle dorsiflexion ≥0° with full knee extension. Exclusion criteria are: (1) subarachnoid haemorrhage or stroke in the cerebellum or brain stem; (2) severe spasticity of the knee or ankle flexors or extensors (i.e., Modified Ashworth Scale [MAS] ≥ 3); (3) pre-existing lower limb deficits or any other medical co-morbidities that might significantly interfere with gait (indicated by a self-reported maximum walking distance <300 meter or walking duration <6 minutes walking pre stroke); (4) severe cognitive problems or aphasia leading to severely impaired comprehension of test instructions; (5) medical conditions that might lead to inability to comply with the study protocol (e.g., congestive heart failure, chemotherapy, uncontrolled epilepsy, pregnancy, depression or psychotic disorder, etc.); (6) demand-type cardiac pacemaker, defibrillator or electrical implant; (7) metallic implant at the affected lower limb; or (8) present or suspected cancerous lesion at the affected lower limb. Potentially eligible participants receive verbal and detailed written information (see Additional file 3) about the study and are invited to participate. In case of willingness to participate, an intake assessment is performed by a researcher who explains the purpose and procedures of the study and asks for informed consent. The following demographics are recorded for each participant: gender, date of birth, body length, body mass, type of stroke, location of stroke (left, right or both), hemiplegic side (left or right), date of stroke, neglect (tactile and visual present or not), relevant co-morbidities, medication and FAC. Furthermore, the following sensorimotor characteristics of both lower limbs are recorded for each participant: Motricity Index (muscle strength) [27], Brunnström Fugl-Meyer Assessment (motor selectivity) [28], and specific parts of the Erasmus Medical Centre Modified Nottingham Sensory Assessment (tactile and proprioceptive sensation) [29], MAS (muscle tone) [30, 31] and passive range of motion at the hip, knee and ankle (PROM). Strategies for patient retention include sending newsletters, accommodating their schedules when planning follow-up visits, sending reminders of upcoming visits, and providing transport support.

### Randomisation and blinding

Concealed randomisation and allocation is effectuated by an assigned researcher (AB), who is not involved in any patient contact, using a computerised randomisation system. Randomisation takes place stratified by functional walking capacity (dependent gait [FAC 1–2] versus independent gait [FAC 3–5]). Outcome assessors are kept blinded to allocation of the participants during all assessments. Participants are instructed not to reveal their group allocation or therapy content to the assessors. Data will be analysed by an independent statistician. Randomisation will be concealed to the primary researcher until data analysis has been completed.

### Interventions

#### Control group

Participants in the control group will receive regular gait training by a physical therapist and/or movement therapist depending on their needs. Typically, per week, three to eight 30-minute sessions of gait-oriented physical therapy are given on five working days for 6 to 12 weeks. This ‘usual care’ may include individual gait training, gait training in groups, fitness training, sports, and hydrotherapy. Walking aids, orthoses, orthopaedic shoes and medication may all be used, but not lower limb FES. Participants will not be restricted in their activities. Therapists are instructed to document characteristics of the gait training (duration, frequency and content) for each participant in weekly logs.

#### Intervention group

Participants in the intervention group receive the same amount of gait-oriented physical therapy, but gait training is assisted by MFES. Per week, MFES is delivered during one 30-minute session on five working days up to 10 weeks. Physical therapists and movement therapists specifically trained in the use of MFES carry out the gait training. They are instructed to document characteristics of the gait training (duration, frequency, content and intensity of MFES) for each participant in weekly logs. During an initial adaptation period of 4 days, the duration of MFES is gradually increased from 15 minutes (day 1) to 30 minutes (day 4). Thereafter, participants receive 30-minute session of MFES-assisted gait training on each workday.

### Multi-channel functional electrical stimulation device

**Fig. 2:**
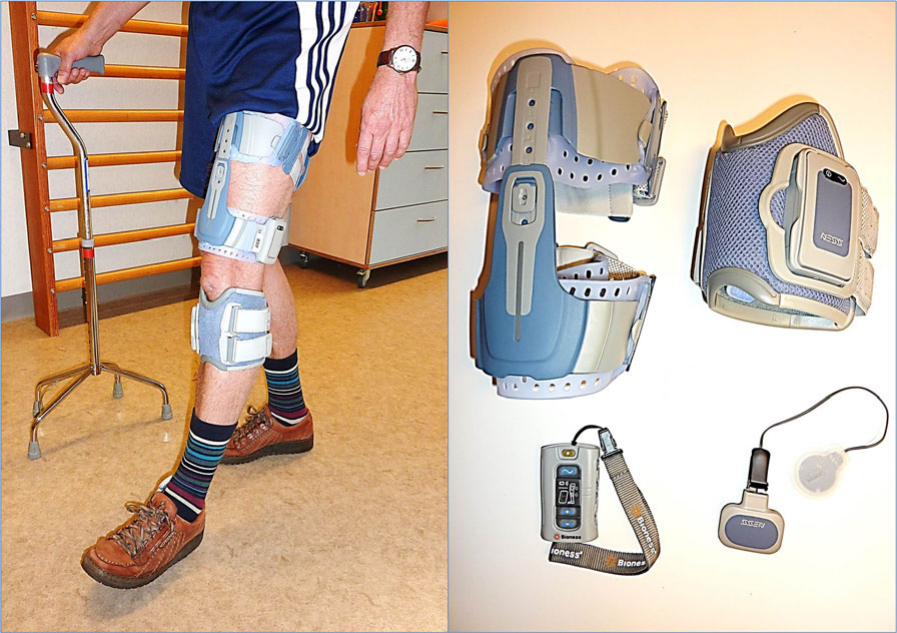
The functional electrical stimulation device including two cuffs, a foot switch, and a control unit

**Fig. 3:**
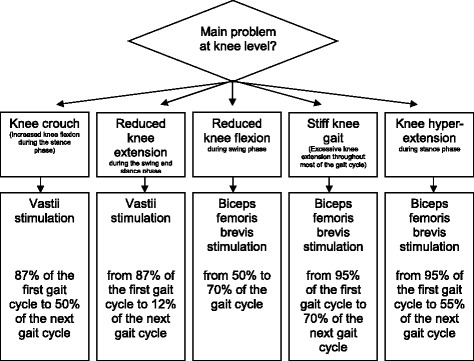
Examples of thigh cuff muscle activation during the gait cycle

The MFES device used in this study (NESS L300^™^ Plus, Bioness, Valencia, CA, USA; CE 0473) delivers electrical pulses during gait to muscles in the affected leg to promote ankle dorsiflexion in combination with knee flexion or extension. The device consists of two cuffs (lower leg and thigh), a foot switch, and a wireless control unit that activates the system by radio frequency signals (Fig. 2). In each cuff two cotton electrodes and a stimulation unit are embedded. The electrodes of the lower leg cuff are located over the common peroneal nerve and the tibialis anterior muscle to elicit ankle dorsiflexion. The electrodes of the thigh cuff are positioned over the vastus medialis muscle to promote knee extension or over the biceps femoris brevis muscle to promote knee flexion. With this configuration, either paretic muscles can be stimulated or spastic muscles antagonised. Figure 3 illustrates some examples of positioning of the thigh cuff and timing of the upper leg stimulation expressed as percentage of the gait cycle [32]. Authorised clinicians are specially trained to fit and set the MFES device. They fit the device at baseline and evaluate the settings of the device every two-and-a-half weeks. A force-sensitive resistor in the foot switch detects the force under the foot. A dynamic gait-tracking algorithm is used to detect whether the foot is on the ground (e.g., initial contact) or in the air (e.g., heel off) by analysing the foot pressure. Average stance and swing phases are calculated by the system and data is transmitted by radio signals to the stimulation unit allowing for the synchronisation of the stimulation in accordance with the timing of gait events (gait mode). During the fitting process, the clinician sets the stimulation parameters (intensity level, phase duration, pulse rate, waveform and maximum duration of stimulation, ramp up, extension and ramp down) for the gait mode with a hand-held computer (personal digital assistant; PDA). The peroneal stimulation starts at ‘heel off’ and terminates at ‘heel contact’. Stimulation can be extended beyond heel contact to control the first rocker. The thigh stimulation – biceps femoris brevis or vastus muscles – can start and end once or twice at any segment in the gait cycle, which is determined by the clinician. Participants who cannot walk without personal assistance receive MFES treatment in the NESS L300^™^ Plus clinician mode (pre-gait and balance training) and gait mode (gait training) during individual physical therapy. The clinician mode is used to manually start and stop stimulation in the thigh and lower leg unit simultaneously. The clinician mode uses the stimulation parameters set for gait mode.

### Outcome measures

#### Primary outcome

The primary outcome to determine efficacy of MFES-assisted gait training is the step length symmetry ratio. Step length during comfortable gait is measured with a spatiotemporal gait analysis system (SGAS) using a laterally placed camera (Panasonic HC-V550 High Definition camera 50 Hertz; Panasonic, Osaka, Japan) and discrete linear transform matrix software designed for this study. Participants walk at comfortable walking speed along a 10-metre walkway until three valid gait trials are collected in which each foot lands within the 1300-millimetre-wide video frame for both sides. The primary condition is walking without shoes and orthosis with minimal use of walking aids. The symmetry ratio is calculated as the difference between the step length of the affected and non-affected leg divided by the mean of the step length of both legs.

#### Secondary outcomes

The SGAS is also used to examine other spatiotemporal parameters (step length, stride length, cadence, stance time symmetry ratio, double support time, and swing/stance time symmetry ratio) for two conditions (walking with and without shoes and orthoses). Furthermore, sagittal and frontal plane video (Basler Scout GigE; Basler AG, Ahrensburg, Germany), electromyography (Mobita and Porti 7 8bt; TMS﻿i, Oldenzaal, the Netherlands) and force plate recordings (OR6-7; AMTI, Watertown, MA, USA) are used to collect kinematic, electromyographic and kinetic data, respectively. One valid gait trial is collected for different conditions (walking with and without shoes, orthoses and walking aids). In addition, at the end of the intervention period, a full three-dimensional gait analysis is performed with an 8-camera VICON MX1.3 motion capture system operating at a sample rate of 100 Hertz (VICON, Oxford, United Kingdom) with two force plates in series recording at 1000 Hertz (OR6-7; AMTI, Watertown, MA, USA) positioned along a 12-metre walkway. Three valid gait trials are collected to register gait width and other kinematic and kinetic parameters that cannot be determined with the SGAS. Walking capacity is assessed with the Functional Gait Assessment (FGA), the FAC and the 10-Meter Walk Test (10MWT), all validated measurement instruments in the stroke population [33]. The FGA is a 10-item test to assess functional gait activities. The FAC is an instrument for categorising gait (in)dependency from ‘no ability to walk or with the help of two or more persons’ (FAC 0) to the ‘ability to walk independently’ (FAC 5). The 10MWT assesses comfortable and maximum walking speed. In this study, only comfortable walking speed will be recorded. Walking capacity is also assessed by a subjective walking capacity recovery score. During each visit the participant is asked to score his or her recovery of walking capacity since the onset of stroke by giving a percentage between 0 % (‘no recovery’) and 100 % (‘full recovery’). Balance control is assessed with the Berg Balance Scale [34–36] and fear of falling with the Falls Efficacy Scale I (FES-I) [37].

### Feasibility

Feasibility of the intervention is evaluated on the basis of compliance with the MFES-assisted gait training and patient satisfaction with this type of training using the MFES device. The following criteria are used: (1) MFES-assisted gait training took place during ≥75 % of all therapy sessions; and (2) patient satisfaction with MFES-assisted gait training was ≥7 on a numeric rating scale from 0 (‘most unsatisfied’) to 10 (‘most satisfied’) assessed at the end of the intervention period. Patient satisfaction with the MFES device is evaluated by a questionnaire designed for this study.

### Sample size

Due to lack of data on effect size, sample size is based on the feasibility of recruitment in one centre with an approximate yearly admission rate of 80 stroke survivors. Using an inclusion period of 3 years and estimating that 25 % of the patients are eligible and willing to participate, the sample size is set at 40 participants (20 in each group).

### Data management and statistical analysis

Data entry takes place by digital and paper case report forms. Personal information of the participants is treated confidentially. Every participant receives an identification number. This number is used on all forms so that no names or other personal information have to be used. Data is saved in a locked cabinet in a locked office and stored digitally in a trial master file for the duration of 15 years. Data quality is guaranteed by random checks of the research database and range checks for data values.

#### Descriptive statistics

Patient characteristics will be described using means, standard deviations, medians, and interquartile ranges (dependent on whether data is normally distributed or not) and percentages. Group comparisons at baseline will be performed with Student’s *t* tests, Mann–Whitney *U* tests and *χ*^2^ tests where appropriate.

#### Primary and secondary analysis

Primary efficacy analysis will be performed on an intention-to-treat basis. In addition, per protocol analyses will be performed. A linear mixed model for repeated measures will be used to analyse differences in the primary outcome and secondary outcomes. A squared time variable will be included to test for a curvilinear recovery curve. The interaction of time by intervention (MFES versus control) assesses whether the slopes of the recovery curves differ between groups. In these analyses both the intercept and the time variable are included as random effects. Group comparisons at the end of the intervention period for the three-dimensional gait analysis parameters and FES-I will be performed with Student’s *t* tests. To assess feasibility of the intervention, the proportion of participants in the intervention group who are compliant with the gait training and who scored ≥7 on the numeric rating scale will be determined. Patient satisfaction with the MFES device will be described. In all analyses, statistical uncertainty will be expressed by means of 95 % confidence intervals. Significance will be set at *p* < 0.05.

### Monitoring and quality assurance

Internal monitoring of the conduct of the study is performed once a year by researchers of the Merem Rehabilitation Centre De Trappenberg and the Academic Medical Centre Amsterdam. The completeness, accuracy, consistency, and procedures are checked according to the monitoring plan. Adverse events (AEs) of the individual participants are reported in the period from signing informed consent (introduction meeting) until the last follow-up meeting. All AEs reported spontaneously by the participant or observed by the primary researcher or staff are recorded. All AEs are followed until they have abated or a stable situation has been reached. Depending on the event, follow-up may require additional tests or medical procedures as indicated, and/or referral to the general physician or a medical specialist. Serious AEs (SAEs) are reported up to the end of study. The sponsor reports the SAEs to the MEC within 15 days after the sponsor has first knowledge of the SAE. SAEs that result in death or appear to be life threatening are reported expedited, i.e. not later than 7 days after the primary researcher has obtained first knowledge of the adverse event. The primary researcher reports the progress of the trial once a year to the MEC.

### Dissemination policy

Trial results are communicated to participants, healthcare professionals, the public, and other relevant groups via newsletters and (inter)national, peer-reviewed journals (Medline database). The results will be presented at relevant (inter)national conferences in rehabilitation and neurology. Furthermore, results will be published on websites of patient societies.

## Discussion

The aim of this study is to evaluate the therapeutic effects of up to 10 weeks of daily MFES-assisted gait training on spatiotemporal parameters, walking capacity, and motor recovery early after stroke. We hypothesise that stroke survivors will benefit from the therapeutic effect of MFES-assisted gait training by larger improvements on spatiotemporal parameters compared to conventional gait training. These data will inform the design of a sufficiently powered (multi-centre) randomised controlled trial. The strength of our study is that we investigate the effects of MFES during functional gait activities. Two out of three studies investigating MFES in the early phase after stroke applied MFES with the patient in a supine position [24, 25]. Moreover, the stimulation periods in the three studies regarding this topic were only 3–4 weeks [23–25]. There is no evidence for the minimum intensity of MFES required to enhance recovery of walking capacity in stroke survivors. Different treatment doses of electrical stimulation have been studied in the past from 15 minutes up to all day long and from once to more sessions a day. The three studies investigating MFES in the early phase after stroke applied MFES for 30–45 minutes and found positive effects on several outcomes [23–25]. In our study, MFES will be applied each workday for minimally 15 minutes to maximally 30 minutes to aim for a feasible protocol in early stroke rehabilitation. Findings from this study will provide insight into the initial effects of MFES-assisted gait training on regaining gait symmetry and several other outcomes in early stroke rehabilitation. The collection of detailed data will generate new knowledge regarding early use of MFES to promote motor and gait recovery in the early phase after stroke. If this study confirms the feasibility and initial efficacy of MFES-assisted gait training, a larger study would be warranted to further determine the effectiveness of this intervention.

## Trial status

At the time of manuscript submission, the enrolment of participants was ongoing at Merem Rehabilitation Centre De Trappenberg, Huizen, the Netherlands.

## Additional files

Addit﻿ional file﻿ 1: SPIRIT ﻿check﻿list. (DOC 127 kb) Additional file 2: Trial registration data. (DOCX 19 kb) Additional file 3: Dutch information letter and informed consent. (PDF 284 kb)

## Abbreviations

10MWT: 10-Meter Walk Test
AEs: adverse events
FAC: Functional Ambulation Categories
FES: functional electrical stimulation
FES-I: Falls Efficacy Scale I
FGA: Functional Gait Assessment
MAS: Modified Ashworth Scale
MEC: Medical Ethics Committee
MFES: multi-channel functional electrical stimulation
PDA: personal digital assistant
PFES: peroneal functional electrical stimulation
PROM: passive range of motion
SAEs: serious adverse events
SGAS: spatiotemporal gait analysis system
