# Supplementary material for: Gait training assisted by multi-channel functional electrical stimulation early after stroke: study protocol for a randomized controlled trial
Source: Trials. 2016 Oct 1;17:477. doi: 10.1186/s13063-016-1604-x (PMC5045622; doi:10.1186/s13063-016-1604-x)
Supplement: Additional file 2: — Trial registration data. (DOCX 19 kb) [file 13063_2016_1604_MOESM2_ESM.docx]

| **Trial registration data** | |
| --- | --- |
| **Data category** | **Information** |
| Primary registry and trial identifying number | www.trialregister.nl, number NTR4762 |
| Date of registration in primary registry | 28 August 2014 |
| Secondary identifying numbers | ABR NL50002.018.14 |
| Source(s) of monetary or material support | Merem Rehabilitation centre De Trappenberg, Huizen, the Netherlands |
| Primary sponsor | Merem Rehabilitation centre De Trappenberg, Huizen, the Netherlands |
| Secondary sponsor(s) | Academic Medical Centre Amsterdam, the Netherlands |
| Contact for public queries | Maijke van Bloemendaal, PT MSc [mbloemendaal@trappenberg.merem.nl]  Merem Rehabilitation centre De Trappenberg, Huizen, the Netherlands |
| Contact for scientific queries | Anita Beelen, PhD [j.a.beelen@amc.uva.nl]  Academic Medical Centre Amsterdam, the Netherlands |
| Public title | Gait therapy assisted by multi-channel functional electrical stimulation in early stroke rehabilitation:  a proof-of-principle RCT |
| Scientific title | Gait therapy assisted by multi-channel functional electrical stimulation in early stroke rehabilitation:  a proof-of-principle RCT |
| Countries of recruitment | The Netherlands |
| Health condition(s) or problem(s) studied | Gait deficits after stroke |
| Intervention(s) | Intervention group: Maximally ten weeks, five days per week, one 30-minute gait therapy session a day (usual care) in which gait is assisted by functional electrical stimulation with a multi-channel device (NESS L300™ Plus) |
|  | Control group: usual care |
| Key inclusion and exclusion criteria | Inclusion criteria: clinical diagnosis of stroke; in the sub-acute stage of stroke (within 31 days since stroke onset); age between 18 and 80 years old; referred to inpatient rehabilitation; medically stable and able to follow an intensive rehabilitation program; indication for gait training; sufficient capacity to stand in-between parallel bars with or without physical assistance and able to walk with aids and physical assistance from one physical therapist; passive range of motion ankle dorsiflexion of at least 0 degrees at full knee extension |
|  | Exclusion criteria: subarachnoid haemorrhage or stroke in the cerebellum or brain stem; severe spasticity of the knee or ankle flexors or extensors; pre-existing lower limb deficits or any other medical co-morbidities that might significantly interfere with gait; severe cognitive problems or aphasia leading to severely impaired comprehension of test instructions; medical conditions that might lead to inability to comply with the study protocol; demand-type cardiac pacemaker, defibrillator or electrical implant; metallic implant at the affected lower limb; or present or suspected cancerous lesion at the affected lower limb |
| Study type | Interventional |
|  | Allocation: randomized intervention model. Parallel assignment masking: single blind (investigator and outcomes assessors) |
|  | Primary purpose: enhancing gait recovery after stroke |
|  | Phase III |
| Date of first enrolment | January 2015 |
| Target sample size | 40 |
| Recruitment status | Open: patient inclusion |
| Primary outcome(s) | Gait symmetry (step length symmetry ratio) |
| Key secondary outcomes | Spatiotemporal parameters (stance time, swing time, double support, swing:stance time, ratios, step length, stride length), kinematics, kinetics, functional gait, gait speed, Patient subjective gait recovery score (percentage), balance, fear of falling, patient satisfaction with the gait training and with the MFES device, and lower limb muscle strength and selectivity, passive range of motion, sensibility and spasticity |
