# Supplementary material for: Gait training assisted by multi-channel functional electrical stimulation early after stroke: study protocol for a randomized controlled trial
Source: Trials. 2016 Oct 1;17:477. doi: 10.1186/s13063-016-1604-x (PMC5045622; doi:10.1186/s13063-016-1604-x)
Supplement: Additional file 3: — Dutch information letter and informed consent. (PDF 284 kb) [file 13063_2016_1604_MOESM3_ESM.pdf]

Functionele elektrostimulatie tijdens looptraining in de vroege fase na een beroerte

Revalidatiecentrum De Trappenberg

Officiële titel: 'Gait training Assisted by dual-channel Functional Electrical Stimulation in early Stroke rehabilitation: a proof-of-principle RCT' (GAFESS study)

Geachte heer/mevrouw,

In deze brief vindt u meer informatie over een onderzoek dat in De Trappenberg wordt uitgevoerd. Wij vragen u vriendelijk om mee te doen aan dit onderzoek. U beslist zelf of u wilt meedoen. Voordat u de beslissing neemt, is het belangrijk om meer te weten over het onderzoek. Lees daarom deze informatiebrief rustig door. Lees ook de Algemene brochure medisch-wetenschappelijk onderzoek voor meer algemene informatie. Wij raden u aan deze informatie met een naaste te bespreken.
Hebt u na het lezen van de informatie nog vragen? Dan kunt u terecht bij uw behandelend fysiotherapeut, arts of de onderzoeker van De Trappenberg. Ook is er een onafhankelijke arts die veel weet van het onderzoek. Op bladzijde 3 vindt u contactgegevens.

Wat is het doel van het onderzoek?

In de komende weken krijgt u revalidatiebehandeling met onder andere looptraining. Mensen die niet optimaal hun spieren in kunnen zetten, kunnen mogelijk baat hebben bij functionele elektrostimulatie. Functionele elektrostimulatie (in het vervolg elektrostimulatie genoemd) is het activeren van (verlamde) spieren middels kleine elektrische impulsen ter ondersteuning van normale dagelijkse activiteiten zoals het lopen. Niet bij iedereen treedt herstel op en het effect verschilt van persoon tot persoon. Met dit onderzoek willen wij uitzoeken wat het effect is van looptraining met functionele elektrostimulatie van onder- en bovenbeenspieren op het herstel van de loopvaardigheid van mensen na een beroerte.

Welke behandeling wordt onderzocht?

In dit onderzoek worden twee behandelingen vergeleken. De nieuwe behandeling bestaat uit het toepassen van elektrostimulatie van het aangedane been tijdens looptraining. De effecten hiervan op het lopen worden vergeleken met effecten van standaard looptraining. <u>Als u meedoet, wordt door loting bepaald of u tijdens de looptraining wel of geen elektrostimulatie krijgt.</u>

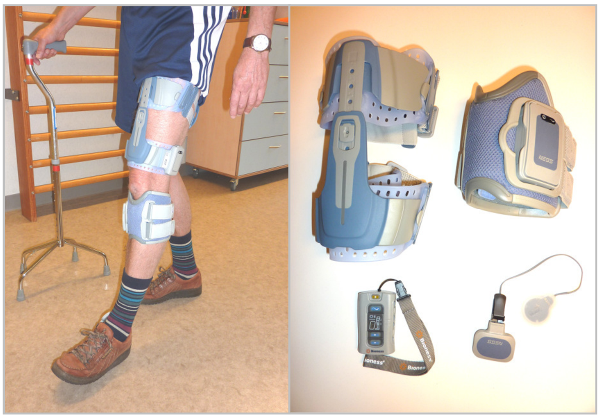

Afbeelding 1: Foto van de elektrostimulator

In dit onderzoek wordt bij de deelnemers met elektrostimulatie zowel het onder- als bovenbeen gestimuleerd met de NESS L300™ Plus elektrostimulator (afbeelding 1). Elektrostimulatie is voelbaar maar niet pijnlijk. De meeste mensen omschrijven elektrostimulatie als een tintelend gevoel. De elektrostimulatie wordt elke werkdag gedurende 30 minuten tijdens de looptraining toegepast onder begeleiding van de fysiotherapeuten van De Trappenberg. De looptraining wijkt verder niet af van de standaard looptraining. De elektrostimulatie tijdens de looptraining duurt maximaal 10 weken.

De elektrostimulatie tijdens de looptraining wordt eerder beëindigd wanneer de deelnemer ontslagen wordt uit de kliniek of wanneer de loopvaardigheid van de deelnemer volledig is hersteld. Daarnaast krijgt de deelnemer de op dat moment gebruikelijke therapieën die deel uit maken van de revalidatiebehandeling.
De deelnemers die standaard looptraining zonder elektrostimulatie krijgen, ontvangen looptraining zoals dat normaalgesproken wordt gegeven in De Trappenberg.

Hoe wordt het onderzoek uitgevoerd?
Om vast te stellen wat de effecten zijn van de behandeling op het herstel van uw loopvaardigheid worden in de komende (maximaal) 24 weken verschillende metingen gedaan. Het gaat om metingen waarbij onder andere loopvaardigheid, balans en spierfunctie worden gemeten en twee vragenlijsten worden afgenomen. Tijdens de (klinische) revalidatiebehandeling worden om de week metingen verricht. Per keer neemt dit ongeveer 1 uur in beslag. De metingen aan het begin van het onderzoek, na 6 weken en (maximaal) 10 weken looptraining zijn uitgebreider en duren ongeveer 2,5 uur. Ook vindt op deze momenten een meting plaats in het looplaboratorium. Hier wordt u gevraagd te staan en te lopen (voor zover dit mogelijk is) op een 10-meter baan waarbij de spieractiviteit wordt gemeten via plakkers (elektroden) op de huid en het staan en lopen op video wordt opgenomen. Per keer neemt dit ongeveer 1,5 uur in beslag. Indien mogelijk vindt er bij de metingen na (maximaal) 10 weken looptraining ook een meting plaats in het geavanceerdere looplaboratorium van de afdeling Revalidatie in het AMC in Amsterdam. Hier zal met speciale camera's de bewegingen van reflecterende bolletjes op de huid tijdens het staan en lopen gemeten worden samen met de spieractiviteit. Deze meting duurt ongeveer 1,5 uur. Na afloop van de looptraining vinden na een maand (1 uur meting) en na drie maanden (2,5 uur meting en 1,5 uur looplaboratorium) nogmaals metingen van loopvaardigheid, balans en spierfunctie plaats. Meer informatie over de metingen kunt u opvragen bij de onderzoeker.

Wat wordt er van u verwacht?
Wij vragen van u om op korte termijn aan te geven bij uw behandelend arts of fysiotherapeut of u wilt deelnemen in verband met starten van de elektrostimulatie tijdens de looptraining in een vroeg stadium (twee tot vier weken) na de beroerte. Wanneer u besluit deel te nemen, vragen wij van u uw tijd en inzet gedurende de metingen en de looptraining. De metingen worden ook na de therapieweken herhaald om na te gaan wat de effecten zijn op lange termijn. De deelnemers die geen elektrostimulatie tijdens looptraining krijgen, mogen in de 10 therapieweken geen elektrostimulatie van het aangedane been ondergaan. Er zijn geen verdere beperkingen die u worden opgelegd.

Wat is meer of anders dan de reguliere behandeling die u krijgt?
De deelnemer die met elektrostimulatie gaat trainen, krijgt in het begin een afspraak met een gespecialiseerd fysiotherapeut om de elektrostimulator aan te meten en in te stellen en eventueel tussentijds om de elektrostimulator bij te stellen.

Wat zijn mogelijke voor- en nadelen van deelname aan dit onderzoek?
Bij deelnemers die met elektrostimulatie trainen kan het lopen mogelijk sneller en beter herstellen maar dat moet dit onderzoek nog uitwijzen. Vooralsnog moet u er van uitgaan dat er geen direct gezondheidsvoordeel is te verwachten. Voor de toekomst kan het onderzoek nuttige gegevens opleveren. De metingen vragen een extra tijdsinvestering van u. Deelnemers die elektrostimulatie krijgen, hebben een kleine kans op huidirritatie op de plaats van de elektroden. Om dit te voorkomen wordt de elektrostimulatie langzaam opgebouwd. Verder zijn er geen nadelige effecten van deze therapie of van de metingen te verwachten.

Wat gebeurt er als u niet wenst deel te nemen aan dit onderzoek?
U beslist zelf of u meedoet aan het onderzoek. Deelname is vrijwillig. Als u besluit niet mee te doen, hoeft u verder niets te doen. U hoeft niets te tekenen. U hoeft ook niet te zeggen waarom u niet wilt meedoen. Als u wel meedoet, kunt u zich altijd bedenken en toch stoppen. Ook tijdens het onderzoek.

Bent u verzekerd wanneer u aan het onderzoek meedoet?
De toetsingscommissie van het AMC heeft dit onderzoek goedgekeurd. Alhoewel het risico op schade gering is, is een verzekering afgesloten die de door het onderzoek veroorzaakte schade van de deelnemer dekt. In de verzekeringsbijlage treft u informatie over de verzekering en adresgegevens van de verzekeraar aan.

Wat gebeurt er met uw gegevens?
Tijdens het onderzoek worden gegevens verzameld over u. Deze gegevens blijven geheim. Uw gegevens krijgen een code en uw naam wordt weggelaten. U zult uw naam dus nooit tegenkomen in een rapport over het onderzoek. Alleen de onderzoeker en medewerkers die direct bij het onderzoek betrokken zijn, weten welke code u heeft. Een paar andere mensen kunnen uw onderzoeksgegevens zien. Deze mensen controleren of het onderzoek goed en betrouwbaar is. Mensen die uw onderzoeksgegevens kunnen zien, zijn het onderzoeksteam, vertegenwoordigers van de opdrachtgever van het onderzoek en vertegenwoordigers van de Inspectie voor de Gezondheidszorg. Uw onderzoeksgegevens worden 15 jaar bewaard na afloop van het onderzoek. Daarvoor geeft u toestemming als u meedoet aan dit onderzoek. Als u dat niet wilt, kunt u niet meedoen aan dit onderzoek.

Wordt uw huisarts en/of behandelend specialist geïnformeerd bij deelname?
Wij laten uw huisarts en/of behandelend specialist schriftelijk weten dat u meedoet aan het onderzoek. U moet hiervoor toestemming geven op het toestemmingsformulier. Als u geen toestemming geeft, kunt u niet meedoen aan het onderzoek.

Zijn er extra kosten of is er een vergoeding wanneer u aan dit onderzoek meedoet?
Tijdens de klinische revalidatieperiode is er geen sprake van extra kosten of een vergoeding voor deelname. Voor de metingen die plaatsvinden na de klinische revalidatieperiode en die niet samenvallen met poliklinische revalidatiebehandeling of plaatsvinden op het AMC, heeft u recht op reiskostenvergoeding (kilometervergoeding gebaseerd op eigen vervoer).

Wilt u verder nog iets weten?
Voor het stellen van vragen en het inwinnen van nadere informatie voor, tijdens en na het onderzoek, kunt u terecht bij Maijke van Bloemendaal, onderzoeker (projectleider).

Maijke van Bloemendaal, promovenda en fysiotherapeut M.Sc. bij De Trappenberg
Telefoonnummer hoofdreceptie De Trappenberg: 035-6929600
E-mailadres: mbloemendaal@trappenberg.merem.nl
Werkzaam: dinsdag t/m donderdag
Website: www.merem.nl/de-trappenberg/kenniscentrum/onderzoek-beroerte

Wanneer zij niet beschikbaar is, kunt u terecht bij Anita Beelen, Hoofd Onderzoek Merem, of uw behandelend arts bij De Trappenberg.

Anita Beelen, Hoofd Onderzoek Merem en senioronderzoeker bij het AMC
Telefoonnummer secretariaat AMC: 020-5664421 of 020-5664049

Wilt u graag een onafhankelijk advies over meedoen aan dit onderzoek? Dan kunt u terecht bij de onafhankelijke deskundige, Mw. M. van Disseldorp, revalidatiearts bij De Trappenberg.

Mw. M. van Disseldorp, revalidatiearts bij De Trappenberg
Telefoonnummer medisch afsprakenbureau: 035-6929650

Als u een probleem of klacht heeft dan kunt u deze aangeven bij de betrokken medewerker(s) en/of bij Maijke van Bloemendaal. Wanneer het niet lukt om dit op te lossen met hen dan kunt u een klacht indienen bij de klachtenfunctionaris. Zie voor meer informatie de brochure 'Klachtenprocedure'. Deze is aanwezig in het folderrek in de middenhal bij de hoofdingang.

Klachtenfunctionaris Merem Behandelcentra
Telefoonnummer hoofdreceptie De Trappenberg: 035-6929600
E-mailadres: klachtenfunctionaris@merem.nl

Bijlagen

- Algemene brochure 'Medisch-wetenschappelijk onderzoek'; en
- Verzekeringsbijlage.

Ondertekening toestemmingsformulier

Functionele elektrostimulatie tijdens looptraining in de vroege fase na een beroerte

ABR nummer NL50002.018.14, versie 5

Ik heb de informatiebrief voor de deelnemer gelezen. Ik kon aanvullende vragen stellen. Mijn vragen zijn genoeg beantwoord. Ik had genoeg tijd om te beslissen of ik meedoe.

Ik weet dat meedoen helemaal vrijwillig is. Ik weet dat ik op ieder moment kan beslissen om toch niet mee te doen. Daarvoor hoef ik geen reden te geven.

Ik geef toestemming om mijn huisarts en/of behandelend specialist te informeren dat ik meedoe aan dit onderzoek.

Ik weet dat sommige mensen mijn gegevens kunnen zien. Die mensen zijn het onderzoeksteam, vertegenwoordigers van de opdrachtgever van het onderzoek en vertegenwoordigers van de Inspectie voor de Gezondheidszorg, zoals vermeld in de informatiebrief.

Ik geef toestemming om mijn gegevens te gebruiken, voor het onderzoek zoals beschreven in de informatiebrief.

Ik geef toestemming om mijn onderzoeksgegevens 15 jaar na afloop van dit onderzoek te bewaren.

Ik wil meedoen aan dit onderzoek.

Naam deelnemer:

Handtekening: Datum : __ / __ / __

Ik verklaar hierbij dat ik deze deelnemer volledig heb geïnformeerd over het genoemde onderzoek.

Als er tijdens het onderzoek informatie bekend wordt die de toestemming van de deelnemer zou kunnen beïnvloeden, dan breng ik hem/haar daarvan tijdig op de hoogte.

Naam onderzoeker (of diens vertegenwoordiger):

Handtekening: Datum: __ / __ / __

Aanvullende informatie is gegeven door (indien van toepassing):

Naam:

Functie:

Handtekening: Datum: __ / __ / __

----

* Doorhalen wat niet van toepassing is.

Verzekeringsbijlage

Overeenkomstig de Wet medisch-wetenschappelijk onderzoek met mensen heeft de AMC Medical Research B.V. voor medisch-wetenschappelijk onderzoek een verzekering afgesloten die door het onderzoek veroorzaakte schade door dood of letsel van de proefpersoon dekt.

- Dit betreft schade die zich tijdens of binnen vier jaar na de deelname aan het onderzoek openbaart en gemeld is binnen vier jaar na beëindiging van de deelname aan het onderzoek.
- Het bedrag waarvoor de verzekering is afgesloten bedraagt € 450.000 per proefpersoon, met een maximum van € 3.500.000 voor het gehele onderzoek en € 5.000.000 voor schade tengevolge van medisch-wetenschappelijk onderzoek die per verzekeringsjaar wordt gemeld.

De verzekering biedt dekking
- voor schade tengevolge van de verwezenlijking van de aan deelname aan het wetenschappelijk onderzoek verbonden risico's waarover men niet schriftelijk is ingelicht;
- voor schade tengevolge van de verwezenlijking van de risico's waarover de deelnemer wél is ingelicht, maar die zich in ernstiger mate voordoet dan is voorzien;
- voor schade tengevolge van de verwezenlijking van de risico's waarover de deelnemer wél is ingelicht, maar die zeer onwaarschijnlijk werd geacht.

De verzekering biedt geen dekking
- voor schade die het gevolg is van het uitblijven van een vermindering van de gezondheidsproblemen van de proefpersoon, dan wel het gevolg is van de verdere verslechtering van de gezondheidsproblemen, indien de deelname aan het wetenschappelijk onderzoek plaatsvindt in het kader van de behandeling van deze gezondheidsproblemen;
- voor schade door aantasting van de gezondheid van de proefpersoon waarvan aannemelijk is dat deze zich ook zou hebben geopenbaard wanneer de proefpersoon niet aan het onderzoek had deelgenomen;
- voor schade tengevolge van deelname aan medisch-wetenschappelijk onderzoek waarbij in de kring van beroepsgenoten gebruikelijke handelingen op het gebied van de geneeskunst met elkaar worden vergeleken en aannemelijk is dat de schade het gevolg is van de toegepaste handelingen;
- voor schade die zich bij een nakomeling van de proefpersoon openbaart als gevolg van een nadelige inwerking van het onderzoek op de proefpersoon of de nakomeling;
- voor schade die het gevolg is van het niet of niet volledig opvolgen van aanwijzingen en instructies door de proefpersoon, indien de proefpersoon daartoe althans in staat is.

De verzekering dekt uitsluitend de schade van natuurlijke personen.
De dekking van specifieke schades en kosten is tot bepaalde bedragen beperkt.

Om aanspraak te kunnen maken op schadevergoeding dient de proefpersoon in geval van vermeende schade als gevolg van het onderzoek dit te melden aan:

Naam verzekeraar: Centramed B.A.
Adres verzekeraar: Postbus 191, 2270 AD Voorburg
Polisnummer: 620.872.806

Voorts wordt de proefpersoon verzocht dienaangaande contact op te nemen met
Anita Beelen, Hoofd Onderzoek Merem en senior onderzoeker bij het AMC,
telefoonnummer Secretariaat Revalidatiegeneeskunde AMC: 020-5664049.
